# Bullous Pemphigoid in a Centenarian Male Simulating Toxic Epidermal Necrolysis

**DOI:** 10.7759/cureus.45037

**Published:** 2023-09-11

**Authors:** Muna Shakhashiro, Taylor R Bradley, Stuart Tobin

**Affiliations:** 1 Dermatology, University of Kentucky College of Medicine, Lexington, USA; 2 Dermatology, Lexington Veterans Affairs (VA) Health Care System, Lexington, USA

**Keywords:** bullous pemphigoid, epidermal necrolysis, bullous skin disease, bullous dermatoses, toxic epidermal necrolysis (ten)

## Abstract

Bullous pemphigoid (BP) is one of the most common autoimmune blistering diseases and classically presents as large, tense bullae. We report a case of BP with toxic epidermal necrolysis (TEN)-like manifestations in a 103-year-old male, the oldest known patient to present with an acute onset of BP. Our patient presented with extensive erosive lesions comprising 12% of the total body surface area, raising suspicion of TEN and Staphylococcal scalded skin syndrome. Detailed clinical, histological, and immunofluorescence analyses were performed, confirming a diagnosis of BP. Atypical presentations of blistering disorders can be a diagnostic challenge and require the use of histologic and direct immunofluorescence testing to distinguish between clinically similar cutaneous diseases. Proper diagnosis is essential to ensure appropriate management and patient care.

## Introduction

Bullous pemphigoid (BP) is one of the most common autoimmune blistering diseases and is characterized by autoantibodies directed against protein components of hemidesmosomes in the epidermis [1]. It primarily affects the elderly population, typically manifesting after the age of 70 [1]. Incidence rates have been shown to increase with age, with the oldest reported case of BP being observed in a 100-year-old woman [2]. BP classically presents with pruritic skin eruptions consisting of large, tense bullae that may evolve into erosions [1]. While BP is traditionally identified by its tense bullae, it has numerous clinical presentations that can mimic other cutaneous diseases [3]. Here, we describe a 103-year-old patient presenting with BP with toxic epidermal necrolysis (TEN)-like skin manifestations.

## Case presentation

**Figure 1:**
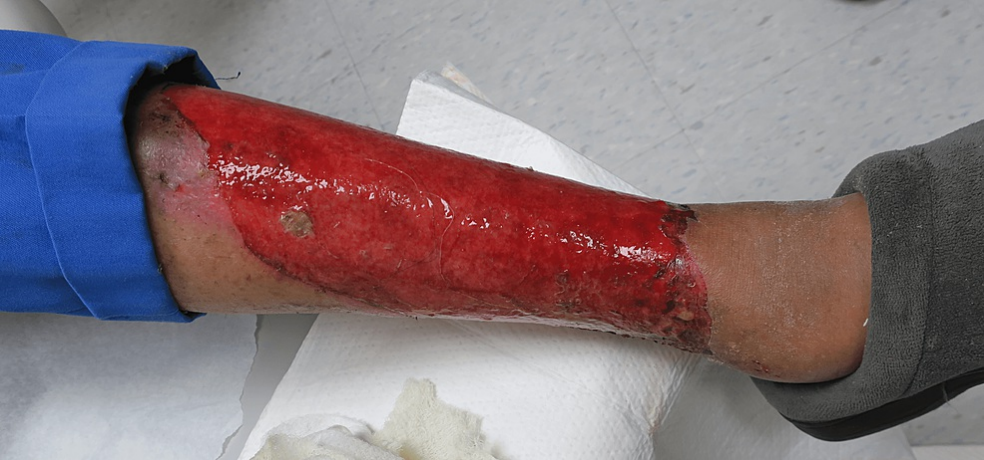
Widespread erosion on the left lower extremity

A 103-year-old African American man with a history of chronic kidney disease and allopurinol use presented to the hospital with two weeks of painful bullous lesions. Physical examination revealed scattered ruptured bullae on the thighs, hands, and arms, with erosions on the lower extremities comprising 12% of the body surface area (BSA) (Figure 1). Based on the extensive erosive lesions, TEN and Staphylococcal scalded skin syndrome (SSSS) were suspected by the admitting staff. Dermatology was consulted.

**Figure 2:**
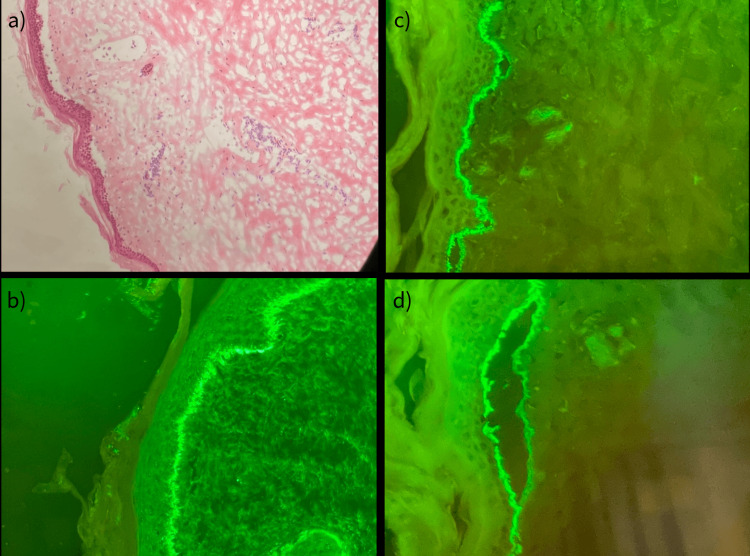
(a) H&E x40 subepidermal blister, (b) DIF linear deposit of IgG, and (c and d) C3 along the basement membrane zone, favoring roof of the bullae H&E: Hematoxylin and eosin stain; DIF: direct immunofluorescence

Two 4 mm punch biopsies were taken from a bullous lesion and perilesional skin. H&E stain revealed an absence of epidermis consistent with TEN; however, direct immunofluorescence (DIF) analysis revealed a linear deposit of IgG and C3 at the basement membrane zone (Figure 2), confirming a diagnosis of bullous pemphigoid. BP230 antibody was also elevated at 11.5 U/ml.

Topical clobetasol and zinc oxide were initiated for treatment for both TEN and BP with skin biopsy and serum antibody results pending, with minimal relief. After the diagnosis of BP was confirmed, treatment was initiated with oral doxycycline (100mg BID), niacinamide (500mg TID), and topical silver sulfadiazine. 

At the eighth month of follow-up, the patient presented with re-epithelialization of the legs and significant clinical improvement. BP230 antibody returned to normal limits.

## Discussion

BP classically presents with diffuse, erythematous pruritic lesions along with development of large tense bullae or fluid-filled blisters [1]. Bullae can evolve into erosions and heal without scarring, and Nikolsky's sign can be absent. In the presented case, an atypical feature was the presence of extensive erosion and flaccid rather than tense bullae. This finding was likely attributable to the patient's aged and atrophic skin. During the initial presentation, the patient’s rash also resembled that of SSSS; however, skin biopsies, the patient’s advanced age, and blood cultures taken during hospital admission quickly ruled out SSSS.

Although several clinical variants of BP exist, TEN-like presentations have rarely been reported [4]. Atypical presentations can be diagnostically challenging, and proper differentiation of BP from TEN is critical given the stark difference in prognosis. TEN is a life-threatening dermatologic emergency presenting with full-thickness epidermal necrosis and sub-epidermal bullae [5]. Hallmark findings include widespread skin erosions with mucosal involvement and a positive Nikolsky's sign. TEN is characterized by epidermal detachment often greater than 10% of the BSA [5]. 

In typical TEN presentations, triggers are identified which often lead to acute symptomatic presentations. In the current case, the patient was taking allopurinol, a known trigger of Stevens-Johnson syndrome/TEN. However, the symptom onset was not associated with initiation of allopurinol use, and the patient sustained continued symptom burden after medication discontinuation, which reduced TEN as a differential diagnosis. Likewise, evidence against TEN included a lack of severe oral lesions or ocular involvement. 

The utilization of histologic and DIF analysis and BP antibody testing can prove beneficial in distinguishing between clinically similar cutaneous disorders. On initial presentation, the patient’s BP 230 antibody testing was elevated and BP 180 titers were normal; however, at the eighth-month follow-up, BP 230 antibody was normal. Histological analysis revealed the absence of epidermis consistent with TEN. However, DIF analysis identified a linear deposit of IgG and C3 at the basement membrane zone (Figure 2), confirming a diagnosis of BP in this 103-year-old patient. 

## Conclusions

We report a case of BP with TEN-like manifestations in a 103-year-old male, the oldest known patient to present with an acute onset of BP. The patient's advanced age contributed to the unique manifestation of extensive erosions and flaccid bullae, deviating from the classic presentation of tense bullae seen in BP. Atypical presentations of blistering disorders can be a diagnostic challenge and require the use of histological analysis and DIF testing to distinguish between clinically similar cutaneous diseases. Comprehensive evaluation and careful consideration of clinical variants are essential for optimizing proper diagnosis and patient outcomes. 

## References

[1] Meijer JM, Terra JB (2016). Cutaneous pemphigoid. Autoimmune Bullous Diseases.

[2] Neufeld R, Portnoy M, Rodstein M (1980). Bullous pemphigoid in a one hundred year old woman. Cutis.

[3] Cozzani E, Gasparini G, Burlando M, Drago F, Parodi A (2015). Atypical presentations of bullous pemphigoid: clinical and immunopathological aspects. Autoimmun Rev.

[4] Qiu C, Shevchenko A, Hsu S (2020). Bullous pemphigoid secondary to pembrolizumab mimicking toxic epidermal necrolysis. JAAD Case Rep.

[5] Schwartz RA, McDonough PH, Lee BW (2013). Toxic epidermal necrolysis: Part I. Introduction, history, classification, clinical features, systemic manifestations, etiology, and immunopathogenesis. J Am Acad Dermatol.

